# An Inverse Problem Involving a Viscous Eikonal Equation with Applications in Electrophysiology

**DOI:** 10.1007/s10013-021-00509-4

**Published:** 2021-06-12

**Authors:** Karl Kunisch, Philip Trautmann

**Affiliations:** 1 Institute for Mathematics and Scientific Computing, University of Graz, Heinrichstrasse 36, A-8010 Graz, Austria; 2 Johann Radon Institute for Computational and Applied Mathematics, Austrian Academy of Science, Linz, Austria

**Keywords:** Inverse problems, Nonlinear elliptic PDEs, Electrophysiology, 35R30, 35J66, 92C30

## Abstract

In this work we discuss the reconstruction of cardiac activation instants based on a viscous Eikonal equation from boundary observations. The problem is formulated as a least squares problem and solved by a projected version of the Levenberg–Marquardt method. Moreover, we analyze the well-posedness of the state equation and derive the gradient of the least squares functional with respect to the activation instants. In the numerical examples we also conduct an experiment in which the location of the activation sites and the activation instants are reconstructed jointly based on an adapted version of the shape gradient method from (J. Math. Biol. 79, 2033–2068, 2019). We are able to reconstruct the activation instants as well as the locations of the activations with high accuracy relative to the noise level.

## Introduction

This work is concerned with an inverse problem in cardiac electrophysiology. In particular, the activation instants of the excitation wave in the myocardium are estimated from the arrival times of the wave at the epicardium. To briefly explain the problem we recall that the electro-physiologic activity of the heart is often modeled using the bidomain equations, whose numerical solution is very expensive. If one is only interested in the activation times *T* of the tissue, the bidomain model can be reduced to the simpler viscous Eikonal equation given, for instance, in the form

1 $$ \left\{ \begin{array}{rll} -\varepsilon\text{div}(M\nabla T)+\sqrt{M\nabla T\cdot \nabla T}&=1&\quad\text{ in }~{{\varOmega}},\\ T&=u_{i}&\quad\text{ on }~{\varGamma}_{i},\quad i=1,\ldots,n\\ \varepsilon \nabla T\cdot n&=0&\quad\text{ on }~{\varGamma}_{N}. \end{array}\right. $$

Here the activation time *T*(*x*) is the time instance when the wave front passes through the point *x* in the domain *Ω* which describes the computational geometry of the heart. The epicardium of the heart is denoted by *Γ*_*N*_ and the boundaries of the activation regions (activation sites) by *Γ*_*i*_. The matrix *M* describes the fiber orientation of the heart tissue and the values $u_{i}\in \mathbb R $ are the activation instants in the activation regions. On the basis of this model we formulate the inverse problem in the following form

2 $$ \min_{u}J(u):=\frac 1 2{\int}_{{\varGamma}_{N}}(T(u)-z)^{2}~\mathrm d x\quad \text{ subject to~(1)}, $$

where *z* is the measured data on the epicardium. Problem (2) constitutes an inverse problem for the activation instants *u*_*i*_. Similar inverse problems involving the Eikonal equation are considered in the context of seismic tomography, see for instance [23]. While in the analysis part we focus on reconstructing the activation instants from measurements of the activation time *T* on the surface of the computational domain *Ω*, in the numerical section we demonstrate that the activation instants and the location of the activation sites can be reconstructed simultaneously.

To briefly comment on the physiological background of this research, we point out that computational models of cardiac function are increasingly considered as a clinical research tool. For the understanding of the driving mechanism of cardiac electro-mechano-fluidic function, the sequence of electrical activations is of key importance. Computer models intended for clinical applications must be parameterized in a patient-specific manner to approximate the electrical activation sequence in a given patient’s heart, which necessitates to solving inverse problems to identify patient specific parameters. Anatomical [5, 18] as well as early experimental mapping studies [6], using *ex vivo* human hearts provided evidence that electrical activation in the left ventricle (LV), i.e. the main pumping chamber that drives blood into the circulatory system, is initiated by the His-Purkinje system [11] at several specific sites of earliest activation (root points) which are located at the endocardial (inner) surface of the LV. In a first approximation it can be assumed that the healthy human LV is activated at these root points by a tri-fascicular conduction system [21] consisting of three major fascicles referred to as anterior, septal and posterior fascicle. Size and location of these patches as well as the corresponding instants of their activation are key determinants shaping the activation sequence of the left ventricle. Since the His-Purkinje system is highly variable in humans, there is significant interest in inverse methods for identifying these sites and activation instants, ideally non-invasively.

As noted above it has become a standard procedure to rely for the mathematical description of the excitation process in the myocardium on various forms of Eikonal equations, see for instance [10, 17, 19]. These are reduced forms of the bidomain equations, a reaction diffusion system which describes the electrical activity of the heart. In [3, Section 5], see also [4], a singular perturbation technique with respect to the thickness of the myocardial wall and the time taken by the excitation wave front to cross the heart wall is carried out to arrive at various models for the Eikonal equation which differ by the nonlinear term. The two versions which are advocated in [3], and for which numerical comparisons are done there, are $|\nabla T|_{M}^{2}$ and $\sqrt {|\nabla T|_{M}^{2}}$. It is stated there that the model involving $\sqrt {|\nabla T|_{M}^{2}}$ is better for wavefront propagation and collision. In the earlier work [15] we have used $|\nabla T|_{M}^{2}$ and solved the inverse shape problem of identifying the centers of the activation regions (spherical subdomains *ω*_*i*_) from epicardial data *z*. An alternative approach for describing the wavefront propagation is based on the use of a function *φ* which at time *t* describes the level set of points for which *φ*(*t*, *x*) = 0 gives the position of the wavefront at that times, see [14]. We refer to [20] for an illuminating summary of wave propagation models involving Eikonal equations.

To briefly outline the paper, first we give a sufficient condition for the well-posedness of the elliptic PDE using the Schauder fixed point theorem and the maximum principle. The activation instants enter the state equation as constant Dirichlet boundary conditions on the surface of the activation regions. Then we calculate the gradient of the least squares cost functional with respect to these activation instants. It can be expressed in terms of the normal derivative of the solution to the adjoint state equation on the surface of activation sites. Therefore we also analyze the well-posedness of the adjoint and linearized state equations. Finally, we solve the least squares problem using the projected Levenberg–Marquardt method, see e.g. [9, 13] for a convergence analysis of this method.

In our first numerical experiments we consider only the reconstruction of the activation instants. In the second numerical example we perform the joint reconstruction of the activation sites and the activation instants. The activation sites are reconstructed by means of an adapted version of the shape gradient method introduced in [15] together with a projected gradient method for the reconstruction of the activation instants. The numerical examples illustrate the feasibility of the approach and are carried out on the 2D unit square with artificial data.

## Problem Statement

Let $U\subset \mathbb {R}^{d}$, with *d* = 2 or *d* = 3 be a bounded domain and *Γ*_*N*_ = *∂U* its boundary. In the physiological context it represents the cardiac domain. Within *U* we consider a family of open subdomains $\{\omega _{i}\}_{i=1}^{n}$ and we set *Γ*_*i*_ = *∂ω*_*i*_. These boundaries constitutes the surface from where the activation spreads. Then we define ${{\varOmega }}=U\setminus \bigcup _{i=1}^{n}\omega _{i}$ which is our mathematical and computational cardiac domain, with boundary $\partial {{\varOmega }}={\varGamma }_{N}\cup \bigcup _{i=1}^{n}{\varGamma }_{i}$. Note that *Ω* is connected but not simply connected. Let us choose a parameter *ε* > 0, and fix $z\in H^{1/2}({\varGamma }_{N})$, which represents the epicardial input data.

With these specifications we consider the following problem:

3 $$ \min_{u\in U_{ad}}J(u)=\frac 1 2{\int}_{{\varGamma}_{N}}(T(x)-z(x))^{2}\mathrm{d} x $$

subject to the viscous Eikonal equation

4 $$ \left\{ \begin{array}{rll} -\varepsilon\text{div}(M\nabla T)+\sqrt{\beta+|\nabla T|_{M}^{2}}&=1&\qquad\text{ in }~{{\varOmega}},\\ T&=u_{i}&\qquad\text{ on }~{\varGamma}_{i},\quad i=1,\ldots,n,\\ \varepsilon M\nabla T\cdot n&=0&\qquad\text{ on }~{\varGamma}_{N}, \end{array}\right. $$

where *β* ∈ [0, 1], *n* is the unit normal on *Γ*_*N*_, and $|\nabla T|_{M}^{2}={ M\nabla T\cdot \nabla T}$. Further $u=\text {col}(u_{1},\dots , u_{n})\in U_{ad}$ which is a bounded, closed, and convex set in $\mathbb {R}^{n}$. The function *T* stands for the activation time, and the matrix *M* models the cardiac conduction velocity.

Compared to the standard viscous Eikonal equation with the nonlinearity $\sqrt {|\nabla T|_{M}^{2}}$ we introduce an additive parameter *β* > 0 in the model considered in (4). It is a mathematical tool which will allow us to guarantee that the control to state mapping *u*↦*T* is differentiable and that the adjoint equation is well-posed. For well-posedness of the optimal control problem in Section 3 the choice *β* = 0 is admissible. In Theorem 2 below we shall argue that the solution *T* of (4) as a function of *β* tends to the solution of (4) with *β* = 0.

## Well Posedness of the Viscous Eikonal Equation and Existence of Optimal Controls

We assume that the boundaries of *Ω* are chosen such that the equation

5 $$ \left\{ \begin{array}{rll} -\varepsilon\text{div}(M\nabla T_{H})&=\tilde f&\qquad\text{in}~{{\varOmega}},\\ T_{H}&=0&\qquad\text{on}~{\varGamma}_{i},\quad i=1,\ldots,n,\\ \varepsilon M\nabla T_{H}\cdot n &=0&\qquad\text{on}~{\varGamma}_{N} \end{array}\right. $$

has a unique solution *T*_*H*_ ∈ *H*^2^(*Ω*) for any $\tilde f\in L^{2}({{\varOmega }})$. Moreover we assume that $M\in W^{1,\infty }({{\varOmega }})^{d\times d}$ and that *M*(*x*)*v* ⋅ *v* ≥ *α*|*v*|^2^ for a.e. *x* ∈*Ω* holds. Further, for any $u\in \mathbb {R}^{n}$ we assume the existence of *g* ∈ *W*^2,6^(*Ω*) with $g|_{{\varGamma }_{i}}=u_{i}$ for *i* = 1, … , *n*, *g* vanishing in a neighbourhood of *Γ*_*N*_, and $\|g\|_{W^{2,6}({{\varOmega }})}\leq c|u|_{\mathbb {R}^{n}}$, with *c* independent of *u*. For example $g={\sum }_{i=1}^{n}u_{i}g_{i}$ can be chosen where the functions *g*_*i*_ are chosen as smooth bump functions which are equal to 1 on $\bar \omega _{i}$, vanish near *Γ*_*N*_ and have the property supp(*g*_*i*_) ∩supp(*g*_*j*_) = *∅* for all *i*, *j* = 1, … , *n*. Moreover for $\tilde T:=T_{H}+g\in H^{2}({{\varOmega }})$, we have $\tilde T|_{{\varGamma }_{i}}=u_{i}$ for all *i* = 1, … , *n* and

$$ \varepsilon {\int}_{{{\varOmega}}} M\nabla T_{H}\cdot \nabla v~\mathrm{d}x={\int}_{{{\varOmega}}} \tilde fv+ \varepsilon \text{div}(M\nabla g)v~\mathrm{d}x $$

for all $v\in V:= {H^{1}_{0}}({{\varOmega }}\cup {\varGamma }_{N})=\{v\in H^{1}({{\varOmega }})~|~v|_{{\varGamma }_{i}}=0,~i=1,\ldots ,n\}$. In the subsequent developments (5) will be used with $\tilde f$ replaced by

$$ -\sqrt{\beta+|\nabla (T_{H} +g)|_{M}^{2}}+ \varepsilon\text{div}(M\nabla g)+1. $$

### **Theorem 1**

*For ε* > 0 *sufficiently large* (4) *has a unique solution*

$$ T\in W^{2,6}({{\varOmega}}). $$

*Moreover there exists a constant c, independent of* $u\in \mathbb {R}^{n}$, *and β* ∈ [0, 1] *such that* $\|T\|_{W^{2,6}} \le c(1+ |u|)$.

### *Proof*

1. Existence: Let $T_{H}\in {H^{1}_{0}}({{\varOmega }}\cup {\varGamma }_{N})$ be fixed. Then we set

$$ f(T_{H})(x):=-\sqrt{\beta+|\nabla(T_{H}(x)+g(x))|_{M}^{2}} $$

with *β* ∈ [0, 1]. Since *T*_*H*_ ∈ *V*, *g* ∈ *W*^2,6^(*Ω*) and $M\in W^{1,\infty }({{\varOmega }})^{d\times d}$ it follows that *f*(*T*_*H*_) ∈ *L*^2^(*Ω*). Now let *w* ∈ *H*^2^(*Ω*) be the unique solution of

6 $$ \left\{ \begin{array}{rll} -\varepsilon\text{div}(M\nabla w)&=f(T_{H})+\varepsilon\text{div}(M\nabla g)+1&\qquad\text{in}~{{\varOmega}},\\ w&=0&\qquad\text{on}~{\varGamma},\\ \varepsilon M\nabla w\cdot n &=0&\qquad\text{on}~{\varGamma}_{N} \end{array}\right. $$

with the estimate

$$ \|w\|_{H^{2}({{\varOmega}})}\leq c(\|f(T_{H})\|_{L^{2}({{\varOmega}})}+|u|+1). $$

Thus we can define the operator $G\colon V \rightarrow H^{2}({{\varOmega }}) \subset V $, *G*: *T*_*H*_↦*w* which satisfies the inequality

7 $$ \|G(T_{H})\|_{H^{2}({{\varOmega}})}\leq c(M,\varepsilon)(\|T_{H}\|_{V}+|u|+1), $$

with *c*(*M*, *ε*) independent of *β* ∈ [0, 1] and *T*_*H*_. In the following we shall utilize Schaefer’s fixed point theorem in order to prove that *G* has a fixed point. At first we prove that $G\colon V\rightarrow V$ is continuous and compact. Let {*T*_*H*, *k*_}_*k*_ ⊂ *V* be a convergent sequence with limit *T*_*H*_ in *V*. We set *w*_*k*_ := *G*(*T*_*H*, *k*_) and have

$$ \sup_{k}\|w_{k}\|_{H^{2}({{\varOmega}})}<\infty, $$

according to (7). The compact embedding of *H*^2^(*Ω*) ∩ *V* in *V* implies the existence of a subsequence {*w*_*k*_} and of a *w* ∈ *V* with $w_{k}\rightarrow w$ in *V*. By taking the limit in the weak formulation of (6) we see that *G*(*T*_*H*_) = *w*. Thus $G\colon V\rightarrow V$ is continuous. A similar argument shows that $G\colon V \rightarrow V$ is compact. In order to apply Schaefer fixed point theorem we have to further show that the set

$$ \{T\in V ~|~T=\lambda G(T)~\text{for some}~0<\lambda\leq1\} $$

is bounded in V. Let *T*_*H*_ ∈ *V* be such that *T*_*H*_ = *λG*(*T*_*H*_) for some 0 < *λ* ≤ 1. Then we have

$$ -\varepsilon\text{div}(M\nabla T_{H})=\lambda(f(T_{H})+\varepsilon \text{div}(M\nabla g)+1)\quad\text{a.e. in }{{\varOmega}}. $$

Multiplying this equation with *T*_*H*_ and integrating over *Ω*, we obtain by Young’s inequality and the fact that 0 < *λ* ≤ 1:

$$ \begin{array}{@{}rcl@{}} \varepsilon\alpha\|\nabla T_{H}\|_{L^{2}({{\varOmega}})}^{2}&\leq&\varepsilon{\int}_{{{\varOmega}}} M\nabla T_{H}\cdot\nabla T_{H}~\mathrm{d}x\\ & =&\lambda{\int}_{{{\varOmega}}}\left( -\sqrt{\beta+|\nabla(T_{H}+g)|_{M}^{2}}+1+\varepsilon \text{div}(M\nabla g)\right)T_{H}~\mathrm{d}x\\ &\leq& \|M\|_{\infty}^{2}\|\nabla T_{H}\|_{L^{2}({{\varOmega}})}^{2}+\frac 32\|T_{H}\|_{L^{2}({{\varOmega}})}^{2} + \frac{\varepsilon^{2}}{2}\|\text{div}(M\nabla g)\|_{L^{2}({{\varOmega}})}^{2}\\ && +\|M\|_{\infty}^{2}\|\nabla g\|_{L^{2}({{\varOmega}})}^{2}+\frac{|{{\varOmega}}|}{2}(\beta+1)\\ &\leq& c(M)\left( \|\nabla T_{H}\|_{L^{2}({{\varOmega}})}^{2}+\varepsilon^{2}|u|^{2}+1+\beta\right), \end{array} $$

with *c*(*M*) independent of *λ* and *ε*. Thus if *ε* is sufficiently large, we have $\|T_{H}\|_{V}\leq \tilde c(M,\varepsilon )(1+|u|)$, for some constant $\tilde c(M,\varepsilon )$ independent of *λ* ∈ (0, 1] and *β* ∈ [0, 1]. Then Schaefer’s fixed point theorem can be applied to *G* and yields the existence of an element *T*_*H*_ ∈ *V* with *G*(*T*_*H*_) = *T*_*H*_ which is a solution of (6). Setting *T* = *T*_*H*_ + *g* we have obtained a solution to (4), for which by (7) we have $|T|_{H^{2}({{\varOmega }})} \le C(M,\varepsilon )$, with *C*(*M*, *ε*) independent of *β* ∈ [0, 1].

Moreover, since ∇*T* ∈ *H*^1^(*Ω*)^*d*^ and thus ∇*T* ∈ *L*^6^(*Ω*)^*d*^, and since also *g* ∈ *W*^2,6^(*Ω*) we have that

$$ -\sqrt{\beta+|\nabla T|_{M}^{2}}+1+\varepsilon\text{div}(M\nabla g)\in L^{6}({{\varOmega}}), $$

and thus

$$ \|T\|_{W^{2,6}({{\varOmega}})} \le \tilde C(M,\varepsilon)(1+|u|) \text { with } \tilde C(M,\varepsilon) \text{ independent of } \beta\in [0, 1]. $$

2. Uniqueness: Let *T*_*i*_ ∈ *W*^2,6^(*Ω*), *i* = 1, 2 be two solutions of (4) and define *δT* = *T*_1_ − *T*_2_. Then *δT* satisfies the equation

8 $$ \left\{ \begin{array}{rll} -\varepsilon\text{div}(M\nabla \delta T)+\sqrt{\beta+|\nabla T_{1}|_{M}^{2}} - \sqrt{\beta+|\nabla T_{2}|_{M}^{2}}&=0&\qquad\text{in}~{{\varOmega}},\\ \delta T&=0&\qquad\text{on}~{\varGamma},\\ \varepsilon\nabla \delta T\cdot n&=0&\qquad\text{on}~{\varGamma}_{N}. \end{array}\right. $$

Let us define for $(x,v) \in {{\varOmega }} \times \mathbb {R}^{d}$ the function

$$ B(x,v):= \left\{\begin{array}{ll} \frac{M(x)v}{\sqrt{\beta+|v|_{M(x)}^{2}}},&\quad v\neq 0,\\ 0,&\quad v=0. \end{array}\right. $$

It is easy to see, that

$$ B(x,\bar v)\cdot(v-\bar v)\leq \sqrt{\beta+|v|_{M}^{2}}-\sqrt{\beta+|\bar v|_{M}^{2}} $$

holds. Indeed, in case $\beta +|\bar v|_{M}^{2} =0$ the inequality is correct by the definition of *B*. Otherwise we have

$$ \begin{array}{@{}rcl@{}} \frac{M\bar v\cdot(v-\bar v)}{\sqrt{\beta+|\bar v|_{M}^{2}}} &=& \frac{M\bar v\cdot v+\beta}{\sqrt{\beta+|\bar v|_{M}^{2}}}-\frac{\beta+|\bar v|_{M}^{2}}{\sqrt{\beta+|\bar v|_{M}^{2}}}\\ &\leq& \frac{\sqrt{\beta+|v|_{M}^{2}}\sqrt{\beta+|\bar v|_{M}^{2}}}{\sqrt{\beta+|\bar v|_{M}^{2}}}-\sqrt{\beta+|\bar v|_{M}^{2}} = \sqrt{\beta+|v|_{M}^{2}}-\sqrt{\beta+|\bar v|_{M}^{2}}. \end{array} $$

Here we have used that $\left (\begin {array}{cc} M & 0 \\ 0 & 1 \end {array} \right )$ defines a scalar product for the vectors $(v,\sqrt {\beta })$. Alternatively we can note that *B*(*x*, *v*) is an element of the subdifferential of the convex function $v\to \sqrt {\beta + |v|_{M}^{2}}$. Thus we have

$$ B(x,\nabla T_{2})\cdot\nabla\delta T\leq \sqrt{\beta+|\nabla T_{1}|_{M}^{2}}-\sqrt{\beta+|\nabla T_{2}|_{M}^{2}}. $$

Consequently,

$$ -\varepsilon\text{div}(M\nabla \delta T)+B(x,\nabla T_{2})\cdot \nabla\delta T\leq 0, $$

where $B(x,\nabla T_{2})\in L^{\infty }({{\varOmega }})^{d}$, since *T*_2_ is an element of *W*^2,6^(*Ω*). Then the maximum principle implies that *δT* ≤ 0 in *Ω*, see [25, Theorem 3.27]. Exchanging the roles of *T*_1_ and *T*_2_ in the above argument leads to *δT* ≥ 0 in *Ω*, and consequently to *δT* = 0, which implies the desired uniqueness. □

This proof is inspired from [8, Section 9.2, Theorem 5]. Henceforth it will be assumed that *ε* is large enough so that the solution to (4) according to Theorem 1 exists.

### **Theorem 2**

*We have*

$$ T_{\beta}\to T_{0}\quad \text{ in }~ H^{2}({{\varOmega}}), $$

*where T*_*β*_ *denotes the solution to* (4) *as a function of β*.

### *Proof*

By Theorem 1 the family $\{T_{H}^{\beta }\}_{\beta \in [0, 1]}$ is bounded in *H*^2^(*Ω*) ∩ *V* and hence there exists a subsequence, denoted in the same manner, and $\hat T_{H} \in H^{2}({{\varOmega }}) \cap V$ such that $T_{H}^{\beta } \rightharpoonup \hat T_{H}$ in *H*^2^(*Ω*) and $T_{H}^{\beta } \to \hat T_{H}$ in *V*. Thus we can pass to the limit in

$$ {\int}_{{{\varOmega}}}\varepsilon M\nabla T_{H}^{\beta}\cdot\nabla \varphi+\sqrt{\beta+|\nabla (T_{H}^{\beta}+g)|_{M}^{2}}\varphi~\mathrm{d}x = {\int}_{{{\varOmega}}}(1+\varepsilon\text{div}(M\nabla g))\varphi~\mathrm{d}x~\text{ for all } \varphi \in V $$

to obtain that

$$ {\int}_{{{\varOmega}}}\varepsilon M \nabla \hat T_{H}\cdot\nabla \varphi +|\nabla (\hat T_{H}+g)|_{M}\varphi~\mathrm{d}x = {\int}_{{{\varOmega}}}(1+\varepsilon\text{div}(M\nabla g)) \varphi~\mathrm{d}x~\text{ for all } \varphi \in V. $$

Moreover, by the trace theorem $\hat T_{H}=0$ on *Γ*_*i*_ for *i* = 1, … , *n*. Now we set $T_{0}=\hat T_{H}+g$. By uniqueness, asserted in Theorem 1 we have $\hat T_{H} = T_{H}$, where *T*_*H*_ is the homogenous solution for *β* = 0 from Theorem 1, and thus the whole family $T_{\beta }=T_{H}^{\beta }+g$ converges to *T*_0_ in *V*. Moreover we have

$$ \begin{array}{@{}rcl@{}} {\int}_{{{\varOmega}}}\varepsilon^{2} \left|\text{div}(M\nabla (T_{\beta} - T_{0}))\right|^{2}~\mathrm{d}x &=& {\int}_{{{\varOmega}}}\varepsilon^{2} \left|\text{div}(M\nabla (T_{H}^{\beta} - T_{H}))\right|^{2}~\mathrm{d}x\\ &=& {\int}_{{{\varOmega}}}\left( \sqrt{\beta+|\nabla T_{\beta}|_{M}^{2}}-|\nabla T_{0}|_{M}\right)^{2}~\mathrm{d}x \to 0 \end{array} $$

for *β* → 0^+^. Since $T_{H}^{\beta }|_{{\varGamma }_{i}},T_{H}|_{{\varGamma }_{i}}=0$ and $\big ({\int \limits }_{{{\varOmega }}}|\text {div}(M\nabla \cdot )|^{2}~\mathrm {d}x\big )^{1/2}$ defines an equivalent norm to the *H*^2^(*Ω*)-norm on *H*^2^(*Ω*) ∩ *V*, the claim follows. □

We close this section by asserting the existence of a solution to (3).

### **Theorem 3**

*There exists an optimal solution to problem* (3).

### *Proof*

Due to boundedness of *U*_*ad*_ and boundedness from below of *J*, there exists a minimizing sequence {*u*_*n*_} in *U*_*ad*_, satisfying

9 $$ \lim_{n\to\infty} J(u_{n})= \inf_{u\in U_{ad}}J(u). $$

Compactness and closedness of *U*_*ad*_ imply the existence of a subsequence $\{u_{n_{k}}\}$ with a limit $\bar u \in U_{ad}$. Let $T(u_{n_{k}})$ denote the solutions to (4) with $u=u_{n_{k}}$. By Theorem 1 the sequence $T(u_{n_{k}})$ is bounded in *W*^2,6^(*Ω*), and hence there exists a subsequence, denoted in the same manner, which converges weakly in *W*^2,6^(*Ω*) and, by Rellich’s compact embedding theorem, strongly in $C^{1}(\bar {{\varOmega }})$ to some $\bar T \in W^{2,6}({{\varOmega }})$. We can now pass to the limit in (4) (with $T=T(u_{n_{k}})$ and $u=u_{n_{k}}$) to obtain that the pair $(\bar u, \bar T)$ satisfies (4). By (9) we have that $\lim _{n\to \infty } J(u_{n_{k}})= J(\bar u) = \min \limits _{u\in U_{ad}}J(u)$. This concludes the proof. □

## Well Posedness of the Linearized and Adjoint State Equation

For the practical realization of (3) the gradient of the cost is an essential tool for iterative solution schemes. For this purpose we investigate in this section the differentiability of the control to solution mapping, and we analyze the adjoint equation which will allow us to obtain a convenient expression for the gradient of the cost. These goals require us to assume *β* > 0, so that the nonlinear term in the state equation is smooth. An alternative procedure might involve proceeding as in [2, 16], at the expense of working with generalized differentiability concepts.

Throughout the rest of the theoretical part of this work *T* ∈ *W*^2,6^(*Ω*) with $M\nabla T\cdot n|_{{\varGamma }_{n}}=0$, and *β* ∈ (0, 1] are assumed. Further *u*, *r* and *h* are chosen arbitrarily in $\mathbb {R}^{n}$, *L*^2^(*Ω*) and $H^{1/2}({\varGamma }_{N})$, respectively.

We analyse the well-posedness of the following equations

10 $$ \left\{ \begin{array}{rll} -\varepsilon\text{div}(M\nabla \delta T)+\frac{M\nabla T\cdot\nabla \delta T}{\sqrt{\beta+|\nabla T|_{M}^{2}}}&=r&\qquad\text{in}~{{\varOmega}},\\ \delta T&= u_{i}&\qquad\text{on}~{\varGamma}_{i},\quad i=1,\ldots,N,\\ \varepsilon M\nabla \delta T\cdot n &=0&\qquad\text{on}~{\varGamma}_{N} \end{array}\right. $$

and

11 $$ \left\{ \begin{array}{rll} -\varepsilon\text{div}(M\nabla \varphi)-\text{div}\left( \frac{M\nabla T}{\sqrt{\beta+|\nabla T|_{M}^{2}}}\varphi\right)&=0&\qquad\text{in}~{{\varOmega}},\\ \varphi&=0&\qquad\text{on}~{\varGamma},\\ \varepsilon M\nabla \varphi\cdot n&=h&\qquad\text{on}~{\varGamma}_{N}. \end{array}\right. $$

For this purpose we define the bilinear form $B\colon V \times V \rightarrow \mathbb {R}$ by

$$ B(v,\varphi):=\varepsilon(M\nabla v,\nabla \varphi)_{L^{2}({{\varOmega}})}+\left( \frac{M\nabla T\cdot \nabla v}{\sqrt{\beta+|\nabla T|_{M}^{2}}},\varphi\right)_{L^{2}({{\varOmega}})} $$

for any *φ*, *v* ∈ *V*. We recall the function *g* ∈ *W*^2,6^(*Ω*) defined in the previous section.

### **Definition 1**

The function *δT* = *v* + *g* ∈ *H*^1^(*Ω*) is called a weak solution of (10) if *v* ∈ *V* solves the variational equation

12 $$ B(v,\varphi)={\int}_{{{\varOmega}}}\left( \varepsilon\text{div}(M\nabla g)-\frac{M\nabla T\cdot\nabla g}{\sqrt{\beta+|\nabla T|_{M}^{2}}}+r\right)\varphi~\mathrm{d}x,\qquad \forall \varphi\in V. $$

Analogously *φ* ∈ *V* is called a weak solution of (11) if it solves the variational equation

$$ B(v,\varphi)={\int}_{{\varGamma}_{N}}gv~\mathrm{d}s,\qquad \forall v\in V. $$

We introduce the operator $\mathcal A\colon V \to V^{\ast }$ and its adjoint $\mathcal A^{\ast }\colon V\to V^{\ast }$ by

$$ \langle \mathcal Av,\varphi\rangle =B(v,\varphi)=\langle v,\mathcal A^{\ast}\varphi\rangle. $$

for all *v*, *φ* ∈ *V*.

### **Proposition 1**

*The operators* $\mathcal A\colon V\to V^{\ast }$ *and* $\mathcal A^{\ast }\colon V \to V^{\ast }$ *are isomorphisms. In particular there exists a constant C*(*M*, *T*, *ε*) *such that*

$$ \|\mathcal A^{-1}\|_{\mathcal L(V^{\ast},V)}=\|\mathcal A^{-\ast}\|_{\mathcal L(V^{\ast}, V)}\leq C(M,T,\varepsilon). $$

### *Proof*

The claims follow from a similar argumentation as in the proof of Proposition 2 in [15] using Garding’s inequality and the weak maximum principle. □

We introduce the space

$$ W=\{v\in H^{2}({{\varOmega}})~|~v|_{{\varGamma}_{i}}\in \mathbb R,~i=1,\ldots,N,~ M\nabla v\cdot n|_{{\varGamma}_{N}}=0\}. $$

The space *W* is a closed subspace of *H*^2^(*Ω*), since the trace as well as the normal trace operator are continuous.

### **Proposition 2**

*Equation* (10) *has a unique weak solution which satisfies δT* ∈ *W and*

13 $$ \|\delta T\|_{H^{2}({{\varOmega}})}\leq C(T,M,\varepsilon,\beta)\left( |u|+\|r\|_{L^{2}({{\varOmega}})}\right). $$

### *Proof*

First we define $L(T,h):=\frac {M\nabla T\cdot \nabla h}{\sqrt {\beta +|\nabla T|_{M}^{2}}}$. We easily see that

$$ \|L(T,h)\|_{L^{2}({{\varOmega}})}\leq C(M)\|\nabla h\|_{L^{2}({{\varOmega}})} $$

holds true. Thus Proposition 1 gives us the existence of *v* ∈ *V* satisfying (12) and we have the estimate

$$ \begin{array}{@{}rcl@{}} \|v\|_{{H^{1}_{0}}({{\varOmega}} \cap{\varGamma}_{N})} &\leq& C(M,T,\varepsilon)\left( \|g\|_{W^{2,6}({{\varOmega}})}+\|L(T,g)\|_{L^{2}({{\varOmega}})}+\|r\|_{L^{2}({{\varOmega}})}\right)\\ &\leq& C(M,T,\varepsilon)\left( \|g\|_{W^{2,6}({{\varOmega}})}+\|r\|_{L^{2}({{\varOmega}})}\right)\\ &\leq& C(M,T,\varepsilon)\left( |u|+\|r\|_{L^{2}({{\varOmega}})}\right). \end{array} $$

This implies that *δT* = *v* + *g* is the unique weak solution of (10). Moving the term *L*(*T*, *v*) to the right-hand side of (10), we conclude with standard elliptic regularity that *δT* ∈ *W* and that (13) holds. □

### **Proposition 3**

*Equation* (11) *has a unique weak solution which satisfies φ* ∈ *H*^2^(*Ω*) ∩ *V* *and*

$$ \|\varphi\|_{H^{2}({{\varOmega}})}\leq C(M,T,\varepsilon)\|h\|_{H^{1/2}({\varGamma}_{N})}. $$

### *Proof*

Proposition 1 implies the existence of a weak solution which satisfies the estimate

$$ \|\varphi\|_{{H^{1}_{0}}({{\varOmega}}\cup{\varGamma}_{N})}\leq C(M,T,\varepsilon)\|h\|_{H^{1/2}({\varGamma}_{N})}. $$

Moving the div-term to the right-hand side of (11) and using

$$ \left\|\text{div}\left( \frac{M\nabla T\varphi}{\sqrt{\beta+|\nabla T|_{M}^{2}}}\right)\right\|_{L^{2}({{\varOmega}})}\leq C(M)\left( \|T\|_{H^{2}({{\varOmega}})}+1\right)\|\varphi\|_{{H^{1}_{0}}({{\varOmega}}\cup{\varGamma}_{N})} $$

which follows from

$$ \text{div}\left( \frac{M\nabla T\varphi}{\sqrt{\beta+\|\nabla T\|_{M}^{2}}}\right)=\text{div}\left( \frac{M\nabla T}{\sqrt{\beta+\|\nabla T\|_{M}^{2}}}\right)\varphi+\frac{M\nabla T\cdot \nabla \varphi}{\sqrt{\beta+\|\nabla T\|_{M}^{2}}} $$

the claim follows from standard elliptic regularity. □

## Derivative of *J*

In this section we characterize the gradient of *J* using the linearized and adjoint state equations. It is an essential tool for every gradient based method, in particular for the numerical realization of problem (2), and will also be used for our numerical examples.

### **Lemma 1**

*There exists a constant C* > 0 *independent of β* > 0 *such that*

$$ \left|\sqrt{\beta+|\nabla T_{1}|_{M}^{2}}-\sqrt{\beta+|\nabla T_{2}|_{M}^{2}}\right|\leq |\nabla(T_{1}-T_{2})|_{M} $$

*holds*.

### *Proof*

There holds

$$ \sqrt{\beta+|\nabla T_{1}|_{M}^{2}}=\left|\left( \beta^{1/2},(M^{1/2}\nabla T_{1})_{1},\ldots,(M^{1/2}\nabla T_{1})_{d}\right)\right|. $$

Using the reverse triangle inequality for |⋅| we get

$$ \begin{array}{@{}rcl@{}} \left|\sqrt{\beta+|\nabla T_{1}|_{M}^{2}} - \sqrt{\beta+|\nabla T_{2}|_{M}^{2}}\right| \!&\leq&\! \left|\left( 0,\left( M^{1/2}\nabla (T_{1}-T_{2})\right)_{1},\ldots,\left( M^{1/2}\nabla (T_{1} - T_{2})\right)_{d}\right)\right|\\ \!&=&\!|\nabla (T_{1}-T_{2})|_{M}. \end{array} $$

□

### **Lemma 2**

*The function f* : *H*^2^(*Ω*) → *L*^2^(*Ω*) *defined by* $f(T):=\sqrt {\beta +|\nabla T|_{M}^{2}}$ *is Frechet differentiable with derivative*

$$ f^{\prime}(T)h=\frac{M\nabla T\cdot \nabla h}{f(T)} $$

*for all β* > 0.

### *Proof*

By multiplication with the conjugate square root we get

$$ \begin{array}{@{}rcl@{}} &&|f(T+h)-f(T)-f^{\prime}(T)h|= \left|\frac{f(T+h)^{2}-f(T)^{2}}{f(T+h)+f(T)}-\frac{M\nabla T\cdot\nabla h}{f(T)}\right|\\ &=&\left|\frac{|\nabla T|_{M}^{2}+2M\nabla T\cdot\nabla h+|\nabla h|_{M}^{2}-|\nabla T|_{M}^{2}}{f(T+h)+f(T)}-\frac{M\nabla T\cdot\nabla h}{f(T)}\right|\\ &=&\left|\frac{(2M\nabla T\cdot \nabla h+|\nabla h|_{M}^{2})f(T)-M\nabla T\cdot\nabla h (f(T+h)+f(T))}{f(T+h)f(T)+f(T)^{2}}\right|\\ &=&\left|\frac{|\nabla h|_{M}^{2}f(T)+M\nabla T\cdot \nabla h (f(T)-f(T+h))}{f(T+h)f(T)+f(T)^{2}}\right|\\ &\leq& \frac{|\nabla h|_{M}^{2}}{f(T)}+\frac{f(T)|\nabla h|^{2}_{M}}{f(T)^{2}}\leq \frac{2}{f(T)}|\nabla h|^{2}_{M}, \end{array} $$

utilizing Lemma 1 and $\|\nabla T\|_{M}\leq \sqrt {\beta +\|\nabla T\|_{M}^{2}}=f(T)$. Then using the embedding *H*^2^(*Ω*)↪*W*^1,4^(*Ω*) and that $f(T)^{-1}\in L^{\infty }({{\varOmega }})$ we get

$$ \begin{array}{@{}rcl@{}} \frac{\|f(T+h)-f(T)-f^{\prime}(T)h\|_{L^{2}({{\varOmega}})}}{\|h\|_{H^{2}({{\varOmega}})}} &\leq& C(M,T)\frac{\|h\|_{W^{1,4}({{\varOmega}})}^{2}}{\|h\|_{H^{2}({{\varOmega}})}}\\ &\leq& C(M,T)\|h\|_{H^{2}({{\varOmega}})}. \end{array} $$

□

### **Theorem 4**

*The operator* $S\colon \mathbb {R}^{N}\to W$, *u*↦*T* *is Frechet differentiable and its derivative* $S^{\prime }(u)\delta u$ *in direction* $\delta u\in \mathbb {R}^{N}$ *is given by the solution δT* ∈ *W of* (10) *with u*_*i*_ = *δu*_*i*_ *for i* = 1, … , *N* *and r* = 0.

### *Proof*

We introduce the mapping $E\colon W\times \mathbb {R}^{N}\to L^{2}({{\varOmega }})\times \mathbb {R}^{N}$ defined by

$$ E(T,u)=\left( \begin{array}{c} -\varepsilon\text{div}(M\nabla T)+\sqrt{\beta+|\nabla T|_{M}^{2}}-1 \\ T|_{{\varGamma}_{1}}-u_{1}\\ {\vdots} \\ T|_{{\varGamma}_{N}}-u_{N} \end{array} \right). $$

Using Lemma 2 it can be argued that *E* is Frechet differentiable. Moreover due to Proposition 2 the operator $D_{T}E(T,u)\colon W\to L^{2}({{\varOmega }})\times \mathbb {R}^{N}$ given by

$$ D_{T}E(T,u)\delta T=\left( \begin{array}{c} -\varepsilon\text{div}(M\nabla \delta T)+\frac{M\nabla T\cdot \nabla \delta T}{\sqrt{\beta+|\nabla T|_{M}^{2}}}\\ \delta T|_{{\varGamma}_{1}}\\ {\vdots} \\ \delta T|_{{\varGamma}_{N}} \end{array} \right) $$

is an isomorphism. Let $(T_{0},u_{0})\in W\times \mathbb {R}^{N}$ such that *E*(*T*_0_, *u*_0_) = 0. Then there exists a neighbourhood $V\subseteq W$ of *T*_0_ and $U\subseteq \mathbb {R}^{N}$ of *u*_0_ and a Frechet differentiable implicit function *S* : *U* → *V*, *u*↦*T* with derivative given by *δT* = *D*_*T*_*E*(*T*, *U*)^− 1^(0, *δu*). Since *u*_0_ is arbitrary, the result follows. □

### **Theorem 5**

*There holds*

$$ \nabla J(u)=S^{\prime}(u)^{\ast}(S(u)-z)=\left( {\int}_{{\varGamma}_{i}}-\varepsilon M\nabla \varphi\cdot n~\mathrm{d}s\right)_{i=1}^{N}, $$

*where φ solves* (11) *for h* = *S*(*u*) − *z*.

### *Proof*

For each $\delta u \in \mathbb {R}^{N}$ we have

$$ DJ(u)\delta u={\int}_{{\varGamma}_{N}}(S(u)-z)S^{\prime}(u)\delta u~\mathrm ds. $$

There holds

$$ \begin{array}{@{}rcl@{}} {\int}_{{\varGamma}_{N}}(S(u)-z) S^{\prime}(u)\delta u ~\mathrm ds&=&{\int}_{{{\varOmega}}}\varepsilon M\nabla \delta T\cdot\nabla \varphi+\frac{M\nabla T\cdot \nabla \delta T}{\sqrt{\beta+|\nabla T|_{M}^{2}}}\varphi~\mathrm dx\\ &&-\sum\limits_{i=1}^{N}{\int}_{{\varGamma}_{i}}\varepsilon M\nabla \varphi\cdot n \delta T~\mathrm ds\\ &=&{\int}_{{{\varOmega}}}\left( -\varepsilon\text{div}(M\nabla \delta T)+\frac{M\nabla T\cdot\nabla \delta T}{\sqrt{\beta+|\nabla T|_{M}^{2}}}\right)\varphi~\mathrm dx\\ &&+\sum\limits_{i=1}^{N}{\int}_{{\varGamma}_{i}}\varepsilon M\nabla \delta T\cdot n \varphi~\mathrm ds\\ &&+{\int}_{{\varGamma}_{N}}\varepsilon M\nabla \delta T\cdot n \varphi~\mathrm ds-{\sum}_{i=1}^{N}{\int}_{{\varGamma}_{i}}\varepsilon M\nabla \varphi\cdot n \delta T~\mathrm ds\\ &=&-\sum\limits_{i=1}^{N}{\int}_{{\varGamma}_{i}}\varepsilon M\nabla \varphi\cdot n \delta u_{i}~\mathrm ds=(S^{\prime}(u)^{\ast}(S(u)-z))\cdot \delta u, \end{array} $$

where $\delta T=S^{\prime }(u)\delta u\in W$ solves (10) with *r* = 0. □

## A Projected Levenberg–Marquardt Method

We solve the inverse problem (3) based on a Levenberg–Marquardt strategy. Let $P_{ad}\colon \mathbb {R}^{d}\to U_{ad}$ be the orthogonal projection on *U*_*ad*_. In particular we iterate

$$ u_{k+1}=P_{ad}(u_{k}+\lambda d), $$

where 0 < *λ* ≤ 1 is the stepsize and *d* solves the problem

$$ \min_{d\in \mathbb{R}^{d}}\frac 12{\int}_{{\varGamma}_{N}}\left( S(u_{k})-z+S^{\prime}(u_{k})d\right)^{2}~\mathrm{d}s + \frac {\alpha_{k}} 2|d|^{2}=j(d). $$

The gradient of *j* is given by

$$ Dj(d)\delta d={\int}_{{\varGamma}_{N}}\left( S(u_{k})-z+S^{\prime}(u_{k})d\right)S^{\prime}(u_{k})\delta d~\mathrm{d}s+\alpha_{k}d\cdot \delta d. $$

Thus we have to solve the equation

$$ \left( S^{\prime}(u_{k})^{\ast} S^{\prime}(u_{k})+\alpha_{k}I\right)d=-S^{\prime}(u_{k})^{\ast}(S(u_{k})-z). $$

Let $\mathcal H(u)$ be the matrix representation of $S^{\prime }(u)^{\ast } S^{\prime }(u)$.

### **Proposition 4**

*The matrix* $\mathcal H(u)$ *is positive definitive and there holds*

$$ S^{\prime}(u_{k})^{\ast} S^{\prime}(u_{k})\delta u=\left( -\varepsilon{\int}_{{\varGamma}_{i}}M\nabla w\cdot n~\mathrm ds\right)_{i=1}^{N} $$

*with w* *is the solution of* (11) *for h* *being the solution* (10) *for* $\delta u\in \mathbb \mathbb {R}^{n}$.

### *Proof*

The formula follows from the exact same calculation as in the proof of Theorem 5, where we replace *T* − *z* by $S^{\prime }(u)\delta u$ and *φ* by *w*. Moreover we have

$$ \mathcal H(u)\delta u\cdot \delta u={\int}_{{\varGamma}_{N}}(S^{\prime}(u)\delta u)^{2}~\mathrm ds\geq 0. $$

The corresponding equality implies $S^{\prime }(u)\delta u=0$ on *Γ*_*N*_. This fact, together with the unique continuation principle [1, 22] and uniqueness of solutions for the linearized state equation (10) imply that *δu* = 0. □

## Numerical Example

In this section we present two numerical examples. In the first one we reconstruct the activation instants using the Levenberg–Marquardt method. As an alternative we could have used the iteratively regularized Gauß–Newton method. For a version with constraints see e.g. [12, 24]. In the second example we jointly reconstruct the positions of the activation regions and the activation instants using a combined shape gradient and projected gradient method.

### Finding the Activation Instants

In this example, the computational domain *U* is given by the unit-square (0, 1) × (0, 1). We consider three activation sites *ω*_*i*_ = *B*_0.1_(*x*_*i*_) whose midpoints are given by *x*_1_ = (0.5, 0.8)^⊤^, *x*_2_ = (0.2, 0.2)^⊤^ and *x*_3_ = (0.8, 0.4)^⊤^. Thus we have ${{\varOmega }}=U\setminus \bigcup _{i=1}^{3}\omega _{i}$. The admissible set is given by $U_{ad}=\{u\in \mathbb R^{3} | u_{i}\geq 0,i=1, 2,3\}$. The observed data is given on the boundary *Γ*_*N*_ of *U*. While the domain is unrealistic from an applications point of view, the fact that the number of activation sites is known is physiologically reasonable. Indeed, early experimental work [21], using ex vivo human hearts provides evidence that electrical activation in the left ventricle is initiated at three discrete sites.

The domain *U* is discretized by 66049 vertices and 131072 triangles, which yields a discretization size of ≈ 4 ⋅ 10^− 3^. The state, linearized state and adjoint variable are approximated by *P*1 finite elements on the mentioned grid using the Fenics toolbox. The nonlinear system of equations for the state variable is solved using Newtons method. The linear equations for the linearized state and the adjoint state are solved using a direct method. Moreover we set *ε* = 0.1 and

$$ M=\left( \begin{array}{cc} \sin(\pi x)+1.1 & 0 \\ 0 & \sin(\pi y)+1.1 \end{array} \right). $$

For the numerical computations we chose *β* = 0 and did not encounter any numerical instabilities due to term $|T(x)|_{M}^{-1}$ in the linearized state and adjoint state equations. In other examples instabilities might arise, which would force us to introduce *β* > 0. The exact activation instants are given by *u*^†^ = (0, 0.1, 0.2)^⊤^. Then observed data *z* is generated by solving the state equation numerically for *T* for *u*^†^, restricting *T* to *Γ*_*N*_ and adding noise *η*. The used perturbance has the form

$$ \eta = \delta \|S(u^{\dagger})\|_{L^{2}({\varGamma}_{N})}\frac{\hat \eta|_{{\varGamma}_{N}}}{\|\hat \eta\|_{L^{2}({\varGamma}_{N})}}, $$

where *δ* ≥ 1 and $\hat \eta $ is a FEM-function with random coefficients on $\bar {{\varOmega }}$. The random coefficients are chosen from a standard normal distribution with zero mean and variance 1. The function $\hat \eta $ is restricted to the boundary *Γ*_*N*_ and denoted by $\hat \eta |_{{\varGamma }_{N}}$. Thus *δ* is the relative noise level. In this example we choose *δ* = 0.1 and *δ* = 10^− 9^.

After discretization of the state and adjoint state variable by linear finite elements, the projected Levenberg–Marquartdt Method can directly be applied. In every step of the iteration the matrix $\mathcal H(u)$ is calculated by solving the linearized state equation and the adjoint equation for all unit vectors resulting in a 3 × 3 matrix. Thus 6 linear PDEs must be solved numerically, see Proposition 4. Moreover the gradient of *J* has to be calculated by solving the nonlinear state equation and the adjoint state equation. So in total 8 PDEs have to be solved per iteration. The nonlinear state equation is solved by the Newton method. Since *β* = 0 the method can be interpreted as a semi smooth Newton method. The iteration is stopped by the discrepancy principle

$$ \|S(u_{K_{\delta}})-z\|_{L^{2}({\varGamma}_{N})}\leq \tau \delta \leq \|S(u_{k})-z\|_{L^{2}({\varGamma}_{N})}\quad\forall~ 0\leq k<K_{\delta} $$

with *τ* > 1, see for instance [7]. In our experiments we choose *τ* = 1.1. The parameter *α*_*k*_ is set to 0.1^*k*^.

In the case *δ* = 0.1 the discrepancy criterium is satisfied after 2 iterations with a final iterate $u_{K_{\delta }}=(0.0004, 0.0981, 0.1860)^{\top }$, the state error $\|S(u_{K_{\delta }})-z\|_{L^{2}({\varGamma }_{N})}=0.0494$ and $|u_{K_{\delta }}-u^{\dagger }|=0.0141$. In the case *δ* = 10^− 9^ the discrepancy criterium is satisfied after 5 iterations with a final iterate $u_{K_{\delta }}=(2.2\cdot 10^{-11}, 0.1, 0.2)^{\top }$, the state error $\|S(u_{K_{\delta }})-z\|_{L^{2}({\varGamma }_{N})}=4.8\cdot 10^{-10}$ and $|u_{K_{\delta }}-u^{\dagger }|=1.1\cdot 10^{-10}$. So we can observe that the activation instants are reconstructed very well relative to the noise level.

### Finding the Activation Instants and Activation Regions

In this section we consider a similar scenario as before. But in addition to the activation instants we also reconstruct the position of the activation regions *ω*_*i*_ by determining the midpoints of *ω*_*i*_. For this purpose we use the shape optimization approach introduced in [15] for the squared version of the Eikonal equation. Here we only modify the formulas developed in that work to fit our state equation. The shape derivative of *J* with respect to a smooth perturbation field *h* with compact support on $U={{\varOmega }}\cup \bigcup _{i=1}^{n}\bar \omega _{i}$ is given by

$$ DJ({{\varOmega}},{\varGamma})h= {\int}_{{{\varOmega}}}S_{1}\colon Dh+S_{0}\cdot h~\mathrm dx $$

for any $h\in \mathcal C_{c}^{\infty }(U,\mathbb {R}^{d})$, where *S*_*i*_, *i* = 0, 1 have the form

$$ \begin{array}{@{}rcl@{}} S_{1}&=&\text{Id}_{\mathbb{R}^{d}}\left( \varepsilon M\nabla T\cdot \nabla \varphi+\left( \|\nabla T\|_{M}-1\right)\varphi\right)\\ &&-\varepsilon(\nabla T\otimes M\nabla \varphi+\nabla \varphi\otimes M\nabla T) -\frac{\nabla T\otimes M\nabla T}{\|\nabla T\|_{M}}\varphi,\\ S_{0}&=&\varepsilon M_{\nabla T}^{\ast}\nabla \varphi+\frac{M_{\nabla T}^{\ast}\nabla T}{2\|\nabla T\|_{M}}\varphi, \end{array} $$

with the outer product *v* ⊗ *w* = *vw*^⊤^ for $v,w\in \mathbb {R}^{d}$, the inner product *G*: *N* = trace(*GN*^⊤^) for $G,N\in \mathbb {R}^{d\times d}$, and

$$ M_{v}h=\left( \sum\limits_{k=1}^{d}DM_{k}v_{k}\right)h, $$

where *M*_*k*_ stands for the *k* th column of *M*. Based on the shape derivative we calculate a perturbation field *h* by solving a linear elasticity equation of the form

$$ {\int}_{U} \gamma Dh \colon Dv+h\cdot v~\mathrm dx=-{\int}_{{{\varOmega}}} S_{1} \colon Dv+S_{0}\cdot v~\mathrm dx, \qquad\forall v\in {H^{1}_{0}}(U,\mathbb{R}^{d}) $$

for *γ* > 0 and thus *h* is a decent direction for *J*. Since we are only interested in the shift of the midpoints *x*_*i*_ of *ω*_*i*_, we average *h* over *ω*_*i*_, *i* = *i*, … , *N*, in order to get a shift of the midpoints. The proposed method is of gradient type and thus we also update the activation instants based on the gradient calculated in Theorem 5. In particular we use a projected gradient method.

In the specific example we choose the exact activation sites as $\omega _{i}^{\dagger }=B_{0.05}(x_{i})$ with $x_{1}^{\dagger }=(0.5, 0.8)^{\top }$, $x_{2}^{\dagger }=(0.2, 0.3)^{\top }$ and $x_{3}^{\dagger }=(0.7, 0.4)^{\top }$. We denote $X^{\dagger }=[x_{1}^{\dagger },x_{2}^{\dagger },x_{3}^{\dagger }]$. The exact activation instants are given by *u*^†^ = (0, 0.1, 0.2).

We start the iteration at the initial points ${x_{1}^{0}}=(0.2, 0.8)^{\top }$, ${x_{2}^{0}}=(0.2, 0.2)^{\top }$ and ${x_{3}^{0}}=(0.8, 0.2)^{\top }$ and initial times *u*^0^ = (0, 0, 0). Relative noise levels are chosen to be *δ* = 0.1, 0.01, 0.001 and the iteration is stopped using the discrepancy criterium.

**Fig. 1:**
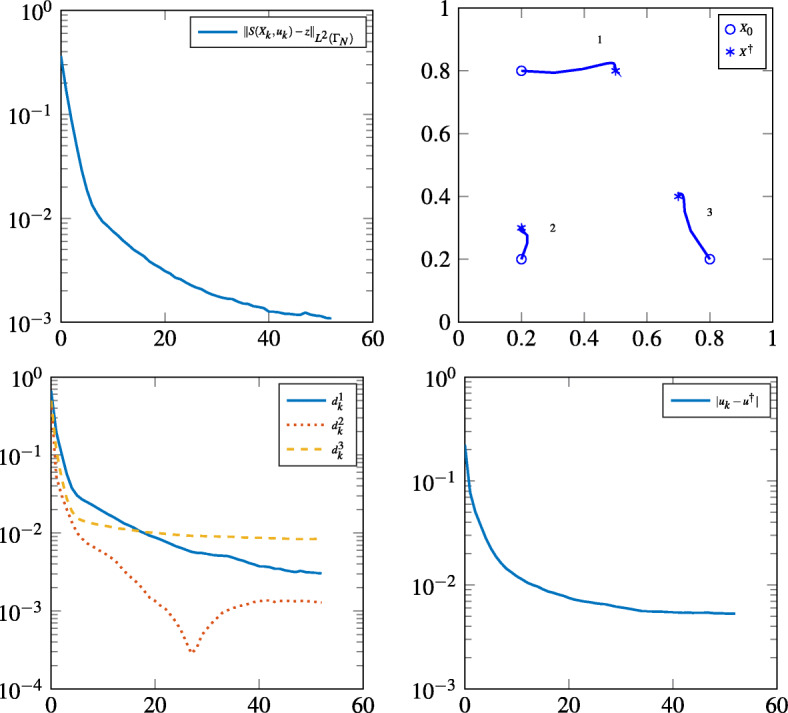
Top left: State error during the iteration; top right: Paths of the midpoints in *U* during the iteration; Bottom left: Distances between *X*_*k*_ and *X*^†^ during the iteration; Bottom right: Error of the activation instants during the iteration

In Table 1 and Fig. 1 we summarize our finding for the three noise levels *δ*. In particular we document the number of iterations *K*_*δ*_ at which the discrepancy criterion is reached, the state error $\|S(X_{K_{\delta }},u_{K_{\delta }})-z\|_{L^{2}({\varGamma }_{N})}$, the distance between reconstructed and exact positions denoted by $d_{K_{\delta }}$ and the reconstruction error $|u_{K_{\delta }}-u^{\dagger }|$. The reconstructed position of the three midpoints as well as the activation instants *u* are given for the respective noise levels by

$$ \begin{array}{@{}rcl@{}} X_{K_{.1}}&=& [(0.396, 0.809),(0.22, 0.275),(0.72, 0.352)],\\ X_{K_{.01}}&=& [(0.496, 0.821),(0.195, 0.296),(0.711, 0.407)],\\ X_{K_{.001}}&=& [(0.499, 0.803),(0.201, 0.301),(0.7, 0.408)] \end{array} $$

as well as

$$ \begin{array}{@{}rcl@{}} u_{.1}&=&(0.038, 0.113, 0.171),\\ u_{.01}&=&(0.011, 0.103, 0.193),\\ u_{.001}&=&(0.003, 0.099, 0.196). \end{array} $$

**Table 1:** Iterations and reconstruction errors for different *δ*

*δ*	*K*_*δ*_	$\|S(X_{K_{\delta }},u_{K_{\delta }})-z\|_{L^{2}({\varGamma }_{N})}$	$d_{K_{\delta }}^{1}$	$d_{K_{\delta }}^{2}$	$d_{K_{\delta }}^{3}$	$|u_{K_{\delta }}-u^{\dagger }|$
0.1	2	0.108	0.104	0.032	0.052	0.049
0.01	9	0.0104	0.021	0.006	0.013	0.013
0.001	52	0.001	0.003	0.001	0.008	0.005

We conclude that the positions can be reconstructed with good quality relative to the noise level. Further tests in the noise free case showed that there is limit until which the state error can be reduced. This is caused by discretization effects.
